# Increased miR-449a expression in colorectal carcinoma tissues is inversely correlated with serum carcinoembryonic antigen

**DOI:** 10.3892/ol.2013.1737

**Published:** 2013-12-06

**Authors:** SHAOHUA CHEN, YUQIAO DAI, XUEMEI ZHANG, DAOZHONG JIN, XIAORONG LI, YI ZHANG

**Affiliations:** 1 Department of General Surgery, The Third Xiangya Hospital of Central South University, Changsha, Hunan 410013, P.R. China; 2 Division of Pharmacology and Toxicology, School of Pharmacy, University of Missouri-Kansas, Kansas City, KS 64108, USA; 3 Department of Gastroenterology, The Third Xiangya Hospital of Central South University, Changsha, Hunan 410013, P.R. China; 4 Department of Basic Medical Sciences, School of Medicine, University of Missouri-Kansas, Kansas City, KS 64108, USA

**Keywords:** miR-449a, colorectal carcinoma, carcinoembryonic antigen

## Abstract

Previously, microRNA-449a (miR-449a) has been shown to be involved in various types of cancer. However, its role in colorectal carcinoma remains unknown. The present study found that miR-449a expression was significantly increased in cultured colorectal carcinoma cells and cancer tissues obtained from 24 patients diagnosed with colorectal carcinoma, compared with the normal colorectal cells and the adjacent non-tumor tissues. The miR-449a expression in carcinoma tissues revealed an inverse correlation with the levels of serum carcinoembryonic antigen (CEA; R^2^=0.88). The increased expression of miR-449a appeared in the patients who exhibited normal serum levels of CEA (<5 ng/ml), but not in those with elevated CEA levels (>5 ng/ml). miR-449a expression increased at an early TNM stage (stage II) and did not change significantly at stages III and IV. These results suggested that miR-449a is involved in the development of colorectal carcinoma and may be a potential prognosis indicator.

## Introduction

Colorectal cancer is the third most common cancer and accounts for 9.4% of cancer cases worldwide (1,2). Its mortality rate among all types of cancer is only below that of lung cancer. Currently, surgery is the only efficient approach to cure colorectal cancer. The cure rate is ≤90% if surgery is performed at the early stage. However, the definitive diagnosis is usually made at the late stage. Late diagnosis and treatment lead to the five-year survival rate of only 40% and ~50% of patients succumb to distant metastasis (1). Thus, in order to improve the cure rate and prognosis of colorectal cancer, it is significantly important for medical researchers to further understand the pathogenic and metastatic mechanisms, and to investigate efficient early diagnosis indicators and new therapy methods.

MicroRNAs (miRNAs) are endogenous non-coding single-stranded RNAs, containing ~22 nt. miRNAs complementarily bind to multiple sites in the 3′ untranslated region of the target mRNA for cleavage or translational repression, and regulate gene expression at the post-transcriptional level. miRNAs are involved in a wide range of physiological and pathophysiological processes, such as cell proliferation, differentiation, apoptosis and development. The dysfunction of miRNAs can cause various diseases, including tumorigenesis. Different miRNAs exert different effects, for example as oncogenes or tumor suppressors, in various types of cancer (3). miRNAs are not only involved in pathogenesis, but are also implicated in the early diagnosis and, therefore, prognosis evaluation of human cancer (4).

microRNA-449a (miR-449a) is an miRNA that has been previously identified in cancer studies. miR-449a is downregulated and shows tumor suppressive effects in various types of cancer, including prostatic carcinoma (5,6); lung (7), liver (8) and gastric (9) cancer; oophoroma (10); and breast (11) and bladder (12) carcinoma. In normal conditions, high levels of miR-449a have been found in testis, lung and trachea tissue (13). In normal colon tissue, a high expression of miR-449a has also been identified, although, the level was relatively lower than that in the other three tissues (13). The expression of miR-449a has also been found in colon cancer-derived cell lines (13,14). However, the expression and clinical significance of miR-449a in colorectal cancer tissues remain unknown.

The present study investigated the expression of miR-449a in human colorectal carcinoma tissues. miR-449a levels were found to be increased in carcinoma tissues, compared with the adjacent non-tumor tissues. The increase of miR-449a expression mainly appeared in patients with normal serum carcinoembryonic antigen (CEA) values, but not in patients with elevated CEA values. The results suggested that miR-449a is an important regulatory factor and potential prognosis indicator in colorectal carcinoma.

## Subjects and methods

### Subjects

In total, 24 patients who received colorectal carcinoma surgery at the Third Xiangya Hospital (Changsha, China) between April 2012 and October 2012 were selected. The diagnosis of colorectal carcinoma was confirmed pathologically. These patients did not receive chemotherapy or radiotherapy. All patients provided signed written informed consent. The study was in strict accordance with National Institutes of Health guidelines and was approved by the ethics committee of The Third Xiangya Hospital of Central South University. Biopsies were obtained from colorectal carcinoma tissues and the adjacent intestinal mucosa (5 cm from the tumor tissues) during surgery. Some tissues were used for pathology to confirm the diagnosis, and a number were used for mRNA extraction and real-time polymerase chain reaction (qPCR).

### Cell cultures

Colorectal carcinoma cells, HT29, SW480, SW620 and HCT116, were purchased from the American Type Culture Collection (Manassas, VA, USA). Normal colonic epithelial cell line, NCM460, was purchased from Incell Corporation, LLC (San Antonio, TX, USA). Cells were grown in RPMI-1640 medium (HyClone; Thermo Fisher Scientific, Waltham, MA, USA), supplemented with heat-inactivated 10% FBS (v/v), 100 U/ml penicillin, 100 μg/ml streptomycin and 1% non-essential amino acids (v/v) (Invitrogen Life Technologies, Carlsbad, CA, USA) and cultured at 37°C in an atmosphere of 5% CO_2_ with a relative humidity of 95%.

### RNA extraction and qPCR

Total RNA was extracted from human tissues or cells using TRIzol reagent (Invitrogen Life Technologies) and the quantity of RNA was measured. The samples, with an A260/A280 ratio of 1.8/2.0, were considered good quality and kept for further use. Total RNA (4 μg) was then reversely transcribed into cDNA using a reverse transcription reagent kit (Invitrogen Life Technologies). The resulting cDNA was detected using an ABI 7500 Real-Time PCR system (Applied Biosystems, Inc., Foster City, CA, USA) with SYBR Green. The small nuclear RNA, U6, was used as an internal control for miR-449a detection (15). The primer sequences for qPCR were as follows: Sense, CTCGCTGGCAGTGTATTGTTAG and antisense, TATCGTTGTACTCCAGACCAAGAC for miR-449a; and sense, CTCGCTTCGGCAGCACA and antisense, AACGCTTCACGAATTTGCGT for U6. After an initial denaturation at 95°C for 3 min, the PCR cycling was performed as follows: 95°C for 12 sec and 65°C for 50 sec. Amplification was performed for 40 cycles and all samples were performed in triplicate. Results were presented as the levels of expression following normalization to U6 using the 2^−ΔCt^ method.

### Serum CEA measurement

Serum CEA levels were measured using ELISA according to the manufacturer’s instructions (no. ab99992; Abcam, Cambridge, UK). The assay employed anti-human CEA antibody coated onto a 96-well plate. Standard or serum samples were pipetted into the wells and CEA present in samples was bound to the wells by the immobilized antibody. The wells were washed and biotinylated anti-human CEA antibody was added. Following the washing of unbound biotinylated antibody, horseradish peroxidase-conjugated streptavidin was pipetted into the wells. The wells were washed again, TMB substrate solution was added and color developed in proportion to the amount of bound CEA. Absorbance value was measured at 450 nm. CEA concentration in serum was achieved according to standard curves.

### Statistical analysis

Data are presented as the mean ± standard error of the mean. Statistical analysis was performed using SPSS 17.0 software (SPSS, Inc., Chicago, IL, USA). The comparison between the two groups was performed by the paired t-test. P<0.05 was considered to indicate a statistically significant difference.

## Results

### PCR primer specificity

**Figure 1:**
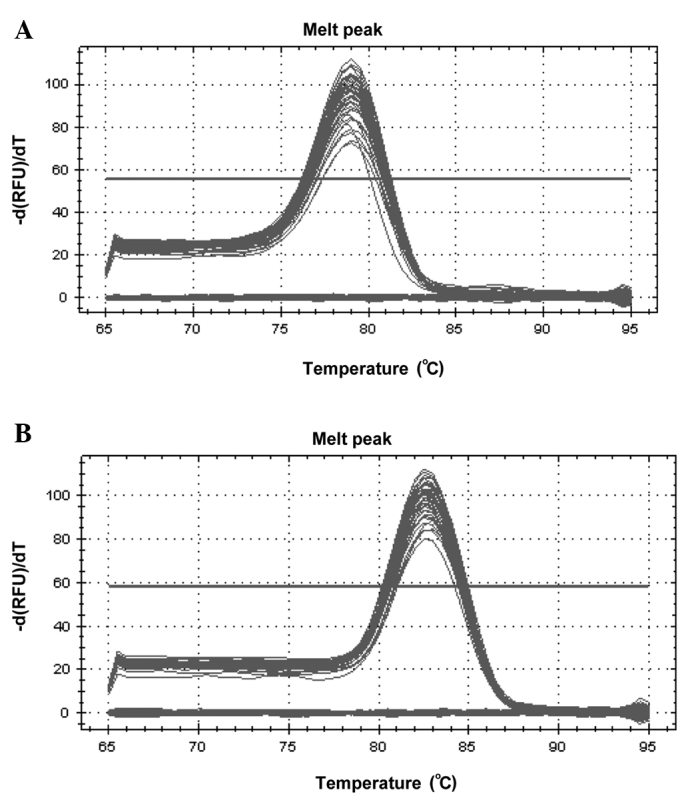
Melting curves of miR-449a and U6 produced at the end of the real-time polymerase chain reaction. The single peaks show the product specificity of (A) miR-449a and (B) U6. miR-449a. miR-449a, microRNA-449a.

Various sequences and sizes of PCR product exhibit different melting temperatures, which produce different melting curves at the end of qPCR. A single peak on the melting curve usually indicates a single reaction product. The melting curves for miR-449a and U6 showed single peaks (Fig. 1), which suggested good specificity for the two primers.

### miR-449a is upregulated in colorectal carcinoma tissues and cells

**Figure 2:**
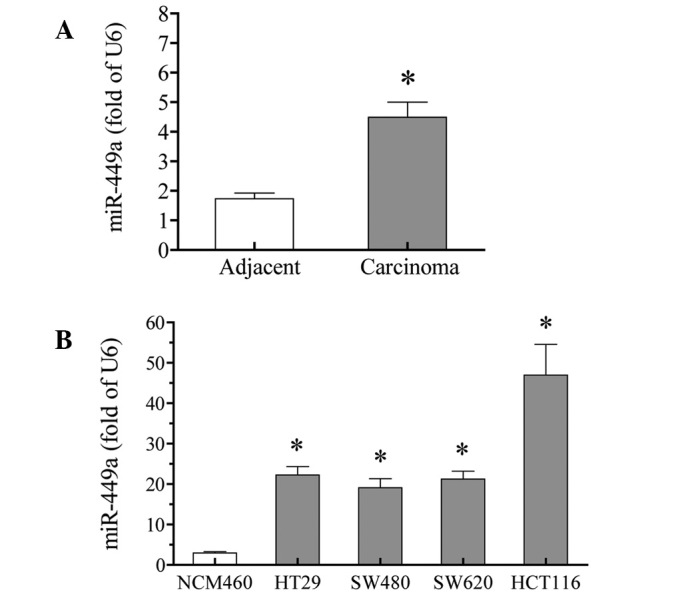
Expression of miR-449a in colorectal tissues and cultured colorectal cells. miR-449a expression was detected in (A) colorectal carcinoma tissues (n=12) and (B) various colorectal carcinoma cells (n=4) by real-time polymerase chain reaction. The results were normalized to the internal control, U6. Adjacent non-tumor tissues (adjacent) and normal colorectal NCM460 cells were used as controls. ^*^P<0.05, vs. the adjacent group and P<0.01, vs. the NCM460 group. miR-449a, microRNA-449a.

miR-449a expression was tested in colorectal carcinoma tissues using qPCR. The results showed that the miR-449a expression was significantly increased in carcinoma tissues, compared with that in adjacent non-tumor tissues. The increase was ≤2.6 fold (P<0.01; Fig. 2A).

To further confirm these results, the expression of miR-449a was examined in cultured colorectal carcinoma cells. All four tested colorectal carcinoma cells, HT29, SW480, SW620 and HCT116, expressed higher levels of miR-449a, compared with the normal colorectal NCM460 cells (P<0.01). Among the four colorectal carcinoma cells, HCT116 cells exhibited the highest expression of miR-449a (>15-fold higher than in NCM460 cells) (Fig. 2B).

### miR-449a negatively correlates with the serum CEA level

**Figure 3:**
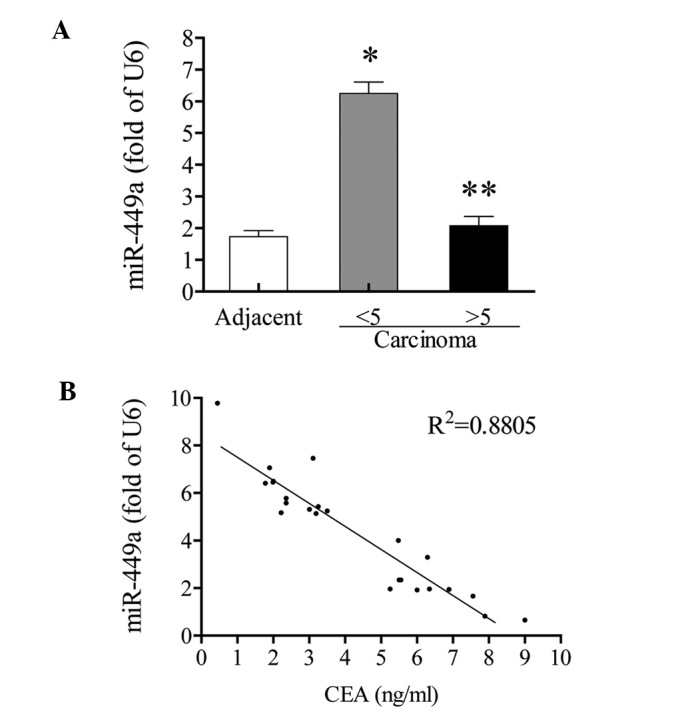
Correlation between miR-449a expression and serum CEA levels. (A) miR-449a expression was detected in colorectal carcinoma and adjacent non-tumor tissues (adjacent) by real-time polymerase chain reaction. The results were normalized to the internal control, U6. The expression of miR-449a in colorectal carcinoma tissues in patients with normal serum CEA (<5 ng/ml; n=13) and abnormal serum CEA (>5 ng/ml; n=11) is shown. (B) The correlation analysis of miR-449a expression in colorectal carcinoma tissues and serum CEA levels is shown. ^*^P<0.01, vs. the adjacent group, ^**^P<0.01, vs. the <5 group and P>0.05, vs. the adjacent group. CEA, carcinoembryonic antigen; miR-449a, microRNA-449a.

Among all 24 patients, 11 patients exhibited abnormal serum CEA levels (>5 ng/ml), while the other 13 patients exhibited normal serum CEA levels (<5 ng/ml). The expression of miR-449a in adjacent non-tumor tissues was not significantly different between patients with normal and abnormal serum CEA levels (data not shown). However, in carcinoma tissues, the increase of miR-449a only appeared in those with normal CEA levels (3.6 fold; P<0.05, vs. the adjacent non-tumor tissues). In patients with abnormal CEA levels, the miR-449a expression was not significantly increased, compared with the adjacent non-tumor tissues (P>0.05; Fig. 3A). In the correlation analysis, the expression of miR-449a in carcinoma tissues showed an inverse correlation with CEA value (R^2^=0.88; Fig. 3B).

### miR-449a expression is independent of TNM stage

**Figure 4:**
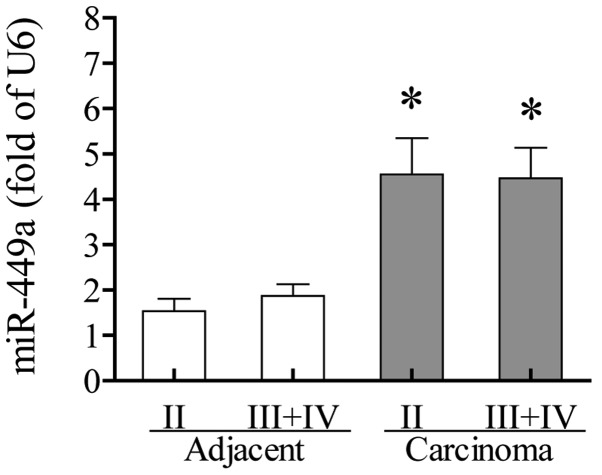
Expression of miR-449a at various TNM stages in colorectal carcinoma tissues. miR-449a expression was detected in colorectal carcinoma and adjacent non-tumor tissues by real-time polymerase chain reaction. The results were normalized to the internal control, U6. miR-449a expression at TNM stages II (n=10) and III+IV (n=14) are shown. ^*^P<0.05, vs. the adjacent group. miR-449a, microRNA-449a.

miR-4409a expression was investigated to identify whether it was found to correlate with TNM stage. The results showed no significant differences between TNM stage II and stages III and IV in the carcinoma tissues and adjacent non-tumor tissues. The increased expression of miR-449a in carcinoma tissues appeared at early TNM stage II and remained at a similarly high level at stages III and IV (Fig. 4). No significant differences were observed among various ages, genders, locations and differentiation degrees (Table I).

## Discussion

miRNA-449a has been previously reported to be downregulated in various types of cancer tissues and may play a tumor-suppressive role. Controversial to these reports, the results of the present study showed that miR-449a was significantly increased in human colorectal carcinoma tissues and cultured colorectal carcinoma cells.

The few previous studies involving miR-449a showed that colon cancer cell lines (V9m, V855, V410 and V478 cells) exhibit a 2-fold higher expression of miR-449 than the normal colon tissues (14). The current study directly compared the expression of miR-449a in colorectal carcinoma tissues and adjacent non-tumor tissues. To the best of our knowledge, the present study is the first to report the elevated expression of miR-449a in human colorectal carcinoma tissues.

miRNAs are a family of non-coding small molecules, which are important in differentiation, proliferation and apoptosis. In addition, miRNAs are important members involved in the tumorigenesis and development of various tumors. As gene silencing factors, miRNAs reveal a complex functional effect. Different miRNAs may exert reverse effects, acting as oncogenes or tumor suppressors in various types of cancer (3,16,17). One miRNA, miR-96, acts as an oncogene in prostate cancer (18) and a tumor suppressor in pancreatic cancer (19). The current study observed the increased expression of miR-449a in colorectal carcinoma tissues and cells. To investigate the possible role of miR-449a in colorectal carcinoma, the correlation between miR-449a expression and serum CEA levels, an important prognosis factor in cancer (including colorectal carcinoma), was compared.

In the current study it was notable that the increased expression of miR-449a in carcinoma tissues only existed in patients with normal serum CEA levels, but not in those with elevated levels of serum CEA. The expression of miR-449a in carcinoma tissues revealed a marked inverse correlation with serum CEA levels. Moreover, with increased CEA levels, the miR-449a was found to decrease towards the levels found in adjacent non-tumor tissues.

A large number of previous studies have shown that higher serum CEA levels predict a worse prognosis in cancer. In rectal cancer, the patients with preoperative serum CEA levels within the normal range have been shown to exhibit a significantly improved prognosis with five-year survival rates of 75.8%, compared with patients with elevated levels who exhibited five-year survival rates of 46.5% (20). In patients with liver and lung metastases of colorectal carcinoma, preoperatively elevated CEA levels are an independent risk factor for low survival (21). Thus, the increase of miR-449a in colorectal carcinoma tissues, as identified in the present study, may indicate a good prognosis.

A previous study in ovarian cancer showed that miR-449a expression was inversely correlated with TNM stage (10). In the present study, the increased miR-449a expression detected at TNM stage II was not significantly different from the expression at stages III and IV. The overexpression of miR-449a may highlight a possible diagnosis candidate for early colorectal carcinoma.

In summary, miR-449a expression is increased in colorectal carcinoma tissues. Its inverse correlation with serum CEA levels suggests that it is a good prognosis indicator. However, its role in colorectal carcinoma (serving as a suppressor/promoter or not) and possible targets, such as CDK6 (11) and HDAC1 (5), require further investigation.

## Figures and Tables

**Table I:** miR-449a expression in colorectal carcinoma tissues of various genders, ages, locations and differentiations.

Groups	miR-449a expression (fold of U6)	P-value
Gender		
Male (n=13)	4.76±0.83	0.575
Female (n=11)	4.17±0.56	
Age, years		
≤60 (n=13)	4.80±0.78	0.530
>60 (n=11)	4.13±0.65	
Location		
Colon (n=15)	4.84±0.65	0.391
Rectum (n=9)	3.91±0.83	
Differentiation		
Poor and moderate (n=16)	4.17±0.56	0.377
Well (n=8)	5.15±1.06	

miR-449a, microRNA-449a.

## References

[1] Corté H, Manceau G, Blons H, Laurent-Puig P (2012). MicroRNA and colorectal cancer. Dig Liver Dis.

[2] Boyle P, Levin B (2008). Colorectal cancer. World Cancer Report 2008.

[3] Farazi TA, Hoell JI, Morozov P, Tuschl T (2013). MicroRNAs in Human Cancer. Adv Exp Med Biol.

[4] Zhu W, Liu X, He J, Chen D, Hunag Y, Zhang YK (2011). Overexpression of members of the microRNA-183 family is a risk factor for lung cancer: a case control study. BMC Cancer.

[5] Noonan EJ, Place RF, Pookot D, Basak S, Whitson JM, Hirata H, Giardina C, Dahiya R (2009). miR-449a targets HDAC-1 and induces growth arrest in prostate cancer. Oncogene.

[6] Noonan EJ, Place RF, Basak S, Pookot D, Li LC (2010). miR-449a causes Rb-dependent cell cycle arrest and senescence in prostate cancer cells. Oncotarget.

[7] Jeon HS, Lee SY, Lee EJ, Yun SC, Cha EJ, Choi E, Na MJ, Park JY, Kang J, Son JW (2012). Combining microRNA-449a/b with a HDAC inhibitor has a synergistic effect on growth arrest in lung cancer. Lung Cancer.

[8] Buurman R, Gürlevik E, Schäffer V, Eilers M, Sandbothe M, Kreipe H, Wilkens L, Schlegelberger B, Kühnel F, Skawran B (2012). Histone deacetylases activate hepatocyte growth factor signaling by repressing microRNA-449 in hepatocellular carcinoma cells. Gastroenterology.

[9] Bou Kheir T, Futoma-Kazmierczak E, Jacobsen A, Krogh A, Bardram L, Hother C, Grønbæk K, Federspiel B, Lund AH, Friis-Hansen L (2011). miR-449 inhibits cell proliferation and is down-regulated in gastric cancer. Mol Cancer.

[10] Zhang Q, He XJ, Ma LP, Li N, Yang J, Cheng YX, Cui H (2011). Expression and significance of microRNAs in the p53 pathway in ovarian cancer cells and serous ovarian cancer tissues. Zhonghua Zhong Liu Za Zhi.

[11] Yang X, Feng M, Jiang X, Wu Z, Li Z, Aau M, Yu Q (2009). miR-449a and miR-449b are direct transcriptional targets of E2F1 and negatively regulate pRb-E2F1 activity through a feedback loop by targeting CDK6 and CDC25A. Genes Dev.

[12] Chen H, Lin YW, Mao YQ, Wu J, Liu YF, Zheng XY, Xie LP (2012). MicroRNA-449a acts as a tumor suppressor in human bladder cancer through the regulation of pocket proteins. Cancer Lett.

[13] Lizé M, Pilarski S, Dobbelstein M (2010). E2F1-inducible microRNA 449a/b suppresses cell proliferation and promotes apoptosis. Cell Death Differ.

[14] Guo C, Sah JF, Beard L, Willson JK, Markowitz SD, Guda K (2008). The noncoding RNA, miR-126, suppresses the growth of neoplastic cells by targeting phosphatidylinositol 3-kinase signaling and is frequently lost in colon cancers. Genes Chromosomes Cancer.

[15] Lardizábal MN, Nocito AL, Daniele SM, Ornella LA, Palatnik JF, Veggi LM (2012). Reference genes for real-time PCR quantification of microRNAs and messenger RNAs in rat models of hepatotoxicity. PLoS One.

[16] Liu M, Tang Q, Qiu M, Lang N, Li M, Zheng Y, Bi F (2011). miR-21 targets the tumor suppressor RhoB and regulates proliferation, invasion and apoptosis in colorectal cancer cells. FEBS Lett.

[17] Watahiki A, Wang Y, Morris J, Dennis K, O’Dwyer HM, Gleave M, Gout PW, Wang Y (2011). MicroRNAs associated with metastatic prostate cancer. PLoS One.

[18] Mihelich BL, Khramtsova EA, Arva N, Vaishnav A, Johnson DN, Giangreco AA, Martens-Uzunova E, Bagasra O, Kajdacsy-Balla A, Nonn L (2011). miR-183-96-182 cluster is overexpressed in prostate tissue and regulates zinc homeostasis in prostate cells. J Biol Chem.

[19] Yu S, Lu Z, Liu C, Meng Y, Ma Y, Zhao W, Liu J, Yu J, Chen J (2010). miRNA-96 suppresses KRAS and functions as a tumor suppressor gene in pancreatic cancer. Cancer Res.

[20] Boras Z, Kondza G, Sisljagić V, Busić Z, Gmajnić R, Istvanić T (2012). Prognostic factors of local recurrence and survival after curative rectal cancer surgery: a single institution experience. Coll Antropol.

[21] Meimarakis G, Angele M, Conrad C, Schauer R, Weidenhagen R, Crispin A, Giessen C, Preissler G, Wiedemann M, Jauch KW (2013). Combined resection of colorectal hepatic-pulmonary metastases shows improved outcome over chemotherapy alone. Langenbecks Arch Surg.

